# Correlation between short-term blood pressure variability parameters with mobil-O-graph pulse wave velocity

**DOI:** 10.1186/s40885-021-00187-x

**Published:** 2022-02-15

**Authors:** Marco Antonio Vieira Silva, Luiz Antonio Pertilli Rodrigues Resende, Mateus Marchiori Vieira, Camila Blanco Ferreira Jajah, Lucas Alves Berzotti, Nicole Cristine Rambourg, Ian Dias de Souza Pierson, João Lucas Carvalho Achkar, Livia Marchiori Vieira, Guilherme Marchiori Moreira, Geisa Ribeiro Borges, Dalmo Correia

**Affiliations:** 1 Division of Tropical Medicine and Infectious Diseases, Department of Internal Medicine, Federal University of the Triângulo Mineiro, 544 Square, Postal Code, Uberaba, 38025-050 Brazil; 2 Division of Cardiology, Department of Internal Medicine, Federal University of the Triângulo Mineiro, Uberaba, Brazil; 3 Santa Casa de São Paulo Medical School, Department of Internal Medicine, São Paulo, Brazil; 4 Federal University of the Triângulo Mineiro, Department of Internal Medicine, Uberaba, Brazil; 5 Uberaba University, Department of Internal Medicine, Uberaba, Brazil; 6 Jundiai Medical College, Department of Internal Medicine, Jundiaí, Brazil; 7 Department of Internal Medicine, Franca University, São Paulo, Brazil

**Keywords:** Arterial stiffness, Short term blood pressure variability, Ambulatory blood pressure monitoring, Hypertension, Pulse wave velocity

## Abstract

**Background:**

Blood pressure variability (BPV) and arterial stiffness show an association with increased cardiovascular events. Evidences demonstrated an association between higher short-term systolic BPV and stiffer arteries. There is no previous study assessed the correlation between BPV and arterial stiffness measured by a Mobil-O-Graph device. We issued to evaluate the correlation between short-term BPV parameters and Mobil-O-Graph pulse wave velocity (PWV) among suspected hypertensive individuals under treatment.

**Methods:**

Mobil-O-Graph device estimated arterial stiffness (oscillometric PWV [oPWV]) in 649 individuals, and they recorded 24-h ambulatory BP; 428 had suspected hypertension and 221 under treatment. We analyzed the correlation between oPWV and measures of BPV: SD of 24 h BP (24-h SD), SD of daytime BP (daytime-SD), and SD of nighttime BP (nighttime-SD), weighted SD of 24-h BP (wSD), coefficient of variation of 24-h BP (CV 24-h) and average real variability (ARV).

**Results:**

Oscillometric PWV showed a positive correlation with all systolic BPV measures, in both groups. Among suspected hypertensives: 24-h SD, r = 0.30; SD daytime-SD, r = 0.34; nighttime-SD, r = 0.16; wSD, r = 0.30; CV 24-h, r = 0.24; ARV, r = 0.22. In the treated individuals: 24-h SD, r = 0.46; daytime-SD, r = 0.47; nighttime-SD, r = 0.35; wSD, r = 0.50; CV 24-h, r = 0.43; ARV, r = 0.37, all *P* < 0.001. Diastolic BPV demonstrated association with some measures of BPV. In suspected hypertensive group: nighttime-SD, r = 0.13; wSD, r = 0.10, both *P* < 0.001. And in treated individuals: daytime-SD, r = 0.23; wSD, r = 0.22; CV 24-h, r = 0.19 (all *P* < 0.001), ARV, r = 0.15 (*P* < 0.05). Systolic daytime-SD in suspected and diastolic CV 24-h in treated group independently predicted oPWV.

**Conclusion:**

We observed a positive and independent correlation between Mobil-O-Graph pulse wave velocity and BPV measures, strong to systolic BPV and weak to diastolic BP.

## Background

Blood pressure (BP) is positively related to vascular and overall mortality, especially throughout middle and old age. Each difference of 20/10 mmHg in systolic BP is associated with more than double the death rate from stroke, ischemic vascular disease, and other vascular causes [1].

Other markers beyond BP can predict cardiovascular (CV) risks, such as arterial stiffness and BPV. Scientific literature has shown how BPV is associated with a higher mortality outcome independent of mean BP or baseline risk of CV events [2, 3].

The output cardiac stroke transmits through the arterial system as a wave. Aortic pulse wave velocity (PWV) is an indirect, well-established index of arterial stiffness. Its speed is inversely related to the arterial wall’s distensibility: the higher the rate, the lower the vascular compliance [4].

Aortic stiffness, expressed as aortic PWV, is a strong predictor for CV risk and all-cause mortality. The higher the patient’s CV risk, the higher the predictive ability of arterial stiffness. Every one m/s increase in aortic PWV corresponds to a risk increase of 14, 15, and 15% in total CV events, CV mortality, and all-cause mortality, respectively. All parameters calculated adjusting for age, sex, and risk factors [5].

The term BPV includes a wide range of BP variations, and they separate into four different groups. The ones that occur over seconds or minutes, known as very short-term BPV. Others that occur within 24 h, known as short-term BPV. Variations between days are known as mid-term or day-to-day BPV. And even so, seasonal variations and changes between clinic visits over months or years, this one, known as long-term BPV [6].

An approach for estimation of short-term BPV consists of performing non-invasive, intermittent 24-h ambulatory blood pressure monitoring (ABPM) at intervals from 15 to 20 min. It allows the straightforward estimation of short-term BPV by calculating 24-h BP standard deviation (SD), daytime SD, and nighttime SD and accounting for its dependence on mean BP levels by calculating the variation of 24-h BP. Despite the simplicity of their calculation, short-term BP variations and the degree of day-night BP reduction influence these indices [7].

Other indices estimate faster BP changes and avoid the interference by day – night BP fluctuations on short-term BPV measures. Average real variability (ARV), computed as the average of the absolute differences between consecutive BP measurements over 24 h, focuses on the sequence of BP readings, thus reflecting short-term, reading-to-reading, within-subject variability in BP levels. The calculation of weighted 24-h BP SD selectively removes the contribution provided by night time BP falls to 24-h SD by weighting the average of daytime and BP SD for the day and night-time periods averaging the SD two-time sub-periods [8].

Aortic intra-arterial PWV is a reliable measure of the global aortic morphological properties, but its invasive assessment makes this approach not feasible in clinical practice. Hence, noninvasive carotid-femoral PWV (cf-PWV) is considered the reference method for its estimation in a clinical setting, given the large number of studies showing cf-PWV a robust independent predictor of total mortality and major CV events [9].

Few previous studies have shown an association between BPV and arterial stiffness. In one of them, short-term systolic BPV showed an independent, moderate, and directly positive relationship with cf-PWV determined noninvasively with the commercially available SphygmoCor system [10].

Mobil-O-Graph device measures oscillometric PWV (oPWV) using cuff oscillometry and pulse wave analysis. Studies in the specialized literature, the oPWV values show acceptable agreement with cf-PWV results from the intra-aortic catheter, Complior, and Artheriograph measurements [11, 12].

Thus, we aimed to study the correlation between six short-term BPV parameters with oPWV among suspected and treated hypertensives individuals.

## Methods

We conducted this cross-sectional study in a specialized center in inner Brazil to diagnose and treat non-communicable diseases. We included in this study all adults older than eighteen with an elevated office BP, whose did not take any hypertensive drug. Here called suspected hypertensives. And people in the treatment of a diagnosed hypertension. Which were referred to perform an ambulatory monitoring blood pressure (AMBP) to confirm a hypertension diagnosis or evaluate uncontrolled hypertension. To define systemic arterial hypertension, we followed the criteria established by 2020 International Society of Hypertension global hypertension practice guidelines [13].

A trained nurse has gotten an informed consent, filled standardized questionnaire with demographic, clinical data, including previous reports of clinical cardiovascular disease (CVD), acute myocardial infarction, acute coronary syndrome, coronary or other arterial revascularization, stroke, transient ischaemic attack, aortic aneurysm, peripheral artery disease and severe chronic kidney disease (CKD). After that, all subjects have measured, weight, height, waist circumference, BP, registered a PWV analysis, and installed an AMBP.

### Blood pressure measurement

After special training the assistant performed measurements of the BP once, with three cuff inflations, utilizing a Microlife equipment BP3AC1-1PC (Onbo Electronic Co, Shenzhen, China). This device has embedded a Microlife Average Mode whose which takes three measurements in succession and calculates the BP average value. Office BP (OBP) measurements followed all recommendations to obtain accurate pressure values [14].

### Pulse wave analysis

Soon in a BP measurement sequence, the individual waits 10 min at rest in a supine position in a quiet room with stable room temperature. Using a Mobil-O-Graph (I.E.M., Stolberg, Germany), the technician performed pulse wave analyses [15, 16]. The individuals performed during the time of pulse wave analysis four BP measurements. The determination of heart rate, peripheral and central pulse pressure, arterial stiffness of Mobil-O-Graph PWV, also called oPWV, followed all Expert Consensus Document’s recommendations on the measurement of Aortic Stiffness 2012 [17].

### Ambulatory monitoring blood pressure

Positioned in a non-dominant arm, an appropriated and equivalent size cuff used to perform BP measurements were fit to record 24 h of ABPM. We utilized a Dyna-Mapa / Mobil-O-Graph-NG monitor (Cardios, São Paulo, Brazil). We took into consideration some requirements for obtaining a satisfactory ABPM. It was acceptable to have at least a minimum of 20 valid daytime (awake) measurements and seven measurements at night (asleep). The measures were obtained every 20 min, throughout the day and every 30 min during the night period. The night period was the time between the individual went to bed and woke up. The times were informed in a diary by the individuals. It was excluded from data analysis all reporters whose not completing all recommendations from AMBP European Guidelines to a quality register [18].

### Parameters of short time BPV

Three short-term measures of systolic and diastolic BP variability resulted directly from AMBP, 24-h SD, daytime-SD, and nighttime-SD. The other three systolic and diastolic BPV measures had their results calculated from AMBP data. The wSD is the mean of daytime and nighttime-SD weighted by the duration (in the number of hours) of day and night time [19**]**. The CV 24-h results from a ratio of 24-h SD by 24-h BP average [20]. And, the ARV calculates the average of absolute changes between consecutive BP readings: ARV = 1 N − 1∑k = 1 N − 1 (BPk + 1 – BPk) where N indicates the number of valid BP measurements, and k is the order of measures [21]. One central parameter of BPV was calculated from the central BP measurements, the SD of central BP.

### Statistical analysies

A database was built using Excel Program and performed the statistical analysis using GraphPad Prism (San Diego, CA) and MedCalc software. The means and SD express the results.

We utilized the D′ Agostino Pearson test to judge the normality of all continuous variables’ distribution. The z scored observed shows satisfactory normal distribution for all variables studied. For comparison of proportions, we applied chi-squared statistics and for means t-test. Correlation analysis explored the association between each BPV measure with oPWV. The Fisher r-to-z transformation compared Pearson coefficients from the same sample [22].. By calculating the partial coefficient, we adjusted the influence of age, sex, and office BP. All clinical variables, BP average, and BPV measures were included in a stepwise regression multiple statistics model to explore their independent influence in oPWV. We formed in regression analysis variables with *P* values under 0.05 and removed P values above 0.1.

## Results

Seven hundred and twelve individuals were eligible. We excluded data from 64 individuals due to a lack of recording of PWV values or an ABPM of acceptable technical quality. So, six hundred and forty-eight individuals had their data analyzed. Among them, 428 had suspected hypertension, and 221 treated hypertension.

Table 1 shows the main clinical characteristics, office, and out-office BP values, variability BP parameters, and oPWV of the whole sample and the two subgroups. The proportion of women and white people was significantly higher in treated than suspected hypertension. Treated hypertensive individuals were older than suspected. And It presented more diabetes, dyslipidemia, abdominal obesity, and clinical CVD with severe CKD. Office BP, oPWV, peripheral pulse pressure, central and peripheral BPV were significantly higher in treated individuals when compared with suspected patients. Conversely, the heart rate was considerably lower. Unlike diastolic, 24-h BP was more elevated in non than in treated hypertensive individuals. There wasn’t any significant difference between the groups to 24-h BP and all diastolic BPV variables.

**Table 1:** The main clinical characteristics, values of BP, BP variability parameters, and pulse wave velocity

Variable	Whole (649)	SH (428)	TH (221)	P Student or x^**2**^
**Clinical characteristic**				
Age (yr)	47.7 ± 14.2	43.4 ± 12.7	56.0 ± 13.2	< 0.0001
White	440 (68.2)	269 (62.8)	171 (77.3)	0.0002
Female sex	320 (49.4)	195 (45.6)	125 (56.6)	0.008
Clinical CVD and CKD	47 (7.3)	11 (2.6)	36 (16.3)	< 0.0001
Diabetes	69 (10.6)	25 (5.8)	44 (19.9)	< 0.0001
Dyslipidemia	188 (29.0)	90 (21.0)	98 (44.3)	< 0.0001
Obesity (kg)	245 (37.8)	166 (38.8)	79 (35.7)	0.44
Smoking	48 (7.4)	30 (10.9)	18 (7.5)	0.16
Abdominal waist at risk	338 (52.0)	206 (48.1)	132 (59.7)	0.005
BMI (kg/m^2^)	28.8 ± 5.8	28.8 ± 5.0	28.7 ± 7.1	0.83
Abdominal waist (cm)	95.6 ± 11.6	95.0 ± 11.9	96.5 ± 11.0	0.11
**BP (mmHg)**				
Systolic office BP	134.9 ± 16.4	133.5 ± 15.2	137.8 ± 18.2	0.0015
Diastolic office BP	86.9 ± 10.9	87.8 ± 10.7	85.1 ± 11.1	0.0027
Systolic 24-h BP	123.3 ± 12.3	123.3 ± 12.0	123.1 ± 12.7	0.84
Diastolic 24-h BP	78.3 ± 10.4	79.1 ± 10.5	76.8 ± 10.2	0.0078
**BP variability (mmHg)**				
SD of 24-h SBP	12.9 ± 3.3	12.7 ± 3.0	13.3 ± 3.6	0.02
SD of 24-h DBP	11.3 ± 3.6	11.3 ± 2.5	11.3 ± 4.9	1.0
SD of daytime SBP	11.9 ± 4.6	11.7 ± 5.0	12.5 ± 3.9	0.03
SD of daytime DBP	9.7 ± 2.5	9.7 ± 2.5	9.7 ± 2.5	1.0
SD of nighttime SBP	9.6 ± 3.3	9.4 ± 3.2	10.0 ± 3.4	0.02
SD of nighttime DBP	8.6 ± 2.7	8.5 ± 2.7	8.9 ± 2.7	0.07
Weighted SD of 24-h SBP	11.3 ± 3.6	11.0 ± 3.8	11.7 ± 3.3	0.02
Weighted SD of 24-h DBP	9.4 ± 2.2	9.4 ± 2.3	9.5 ± 2.1	0,58
Systolic ARV of 24 h	8.9 ± 2.4	8.7 ± 2.2	9.2 ± 2.6	0,01
Diastolic ARV of 24 h	7.7 ± 1.7	7.7 ± 1.7	7.7 ± 1.8	1.0
CV of 24-h SBP	10.5 ± 2.4	10.3 ± 2.2	14.5 ± 3.3	< 0.0001
CV of 24-h DBP	14.6 ± 5.0	10.8 ± 2.5	14.9 ± 7.3	< 0.0001
SD of central SBP	3.7 ± 2.6	3.4 ± 2.4	4.4 ± 2.9	< 0.0001
SD of central DBP	2.7 ± 1.9	2.5 ± 1.7	3.0 ± 2.3	0.012
**Pulse wave analysis**				
Mobil-O-Graph PWV **(**m/s)	7.5 ± 1.7	7.0 ± 1.4	8.5 ± 1.8	< 0.0001
Heart rate (bpm)	70.9 ± 10.9	71.8 ± 10.9	69.3 ± 10.7	0.005
Peripheral pulse pressure	48.3 ± 10.1	47.5 ± 9.3	49.9 ± 11.3	0.004
Central pulse pressure	38.3 ± 8.9	38.0 ± 8.5	38.9 ± 9.5	0.22

**Data are demonstrated as mean ± standard deviation (SD), absolute number and proportion (%).**

BP, blood pressure; SH, suspected hypertension; TH, treated hypertension; CVD, cardiovascular disease; CKD, severe chronic kidney disease; BMI, body mass index; SBP, systolic blood pressure; DBP, diastolic blood pressure; ARV, average real variability; CV, coefficient of variation; PWV, pulse wave velocity.

In bivariate analyses, systolic OBP and 24-h BP showed a positive correlation with PWV. After adjustment for age and sex, the result was kept in the total selected, and both groups. All systolic BPV variables showed a good and straight correlation with oPWV in the whole sample and both groups (Table 2). Among the entire subjects and suspected hypertensives, daytime-SD reached the strongest association, followed by weighted SD of 24-h, SD of 24-h BP, ARV of 24-h, CV of 24-h, nighttime-SD, and SD central BP.

**Table 2:** Correlation between oscillometric pulse wave velocity and measures of systolic BP variability

	Pearson correlation coefficient (r)					
	Whole (649)		SH (428)		TH (221)	
	Univariable	Partial	Univariable	Partial	Univariable	Partial
**BP**		Adjusted for age and sex		Adjusted for age and sex		Adjusted for age and sex
Office BP	0.42^**^	0.51^**^	0.41^**^	0.55^**^	0.44^**^	0.48^**^
24-h BP	0.27^**^	0.43^**^	0.24^**^	0.44^**^	0.35^**^	0.37^**^
**BP variability**		Adjusted for age, sex, and systolic 24-h BP		Adjusted for age, sex, and systolic 24-h BP		Adjusted for age, sex, and systolic 24-h BP
SD of 24-h BP	0.36^**^	0.16^**^	0.30^**^	0.16^**^	0.46^**^	0.10^a)^
SD of daytime BP	0.40^**^	0.20^**^	0.34^**^	0.18^**^	0.47^**^	0.16^*^
SD of nighttime BP	0.24^**^	0.12^**^	0.16^**^	0.11^*^	0.35^**^	0.09^a)^
Weighted SD of 24-h BP	0.39^**^	0.19^**^	0.30^**^	0.17^**^	0.50^**^	0.16^*^
ARV of 24 h	0.33^**^	0.16^**^	0.24^**^	0.12^*^	0.43^**^	0.16^*^
CV of 24-h BP	0.30^**^	0.16^**^	0.22^**^	0.16^**^	0.37^**^	0.10^a)^
SD of central BP	0.23^**^	0.21^**^	0.13^*^	0.18^**^	0.22^**^	0.23^**^

BP, blood pressure; r, Pearson correlation coefficient; SH, suspected hypertension; TH, treated hypertension; SD, standard deviation; ARV, average real variability; CV, coefficient of variation.

*P < 0.05, **P < 0.001, ^a)^ P, not significant

**Fig. 1:**
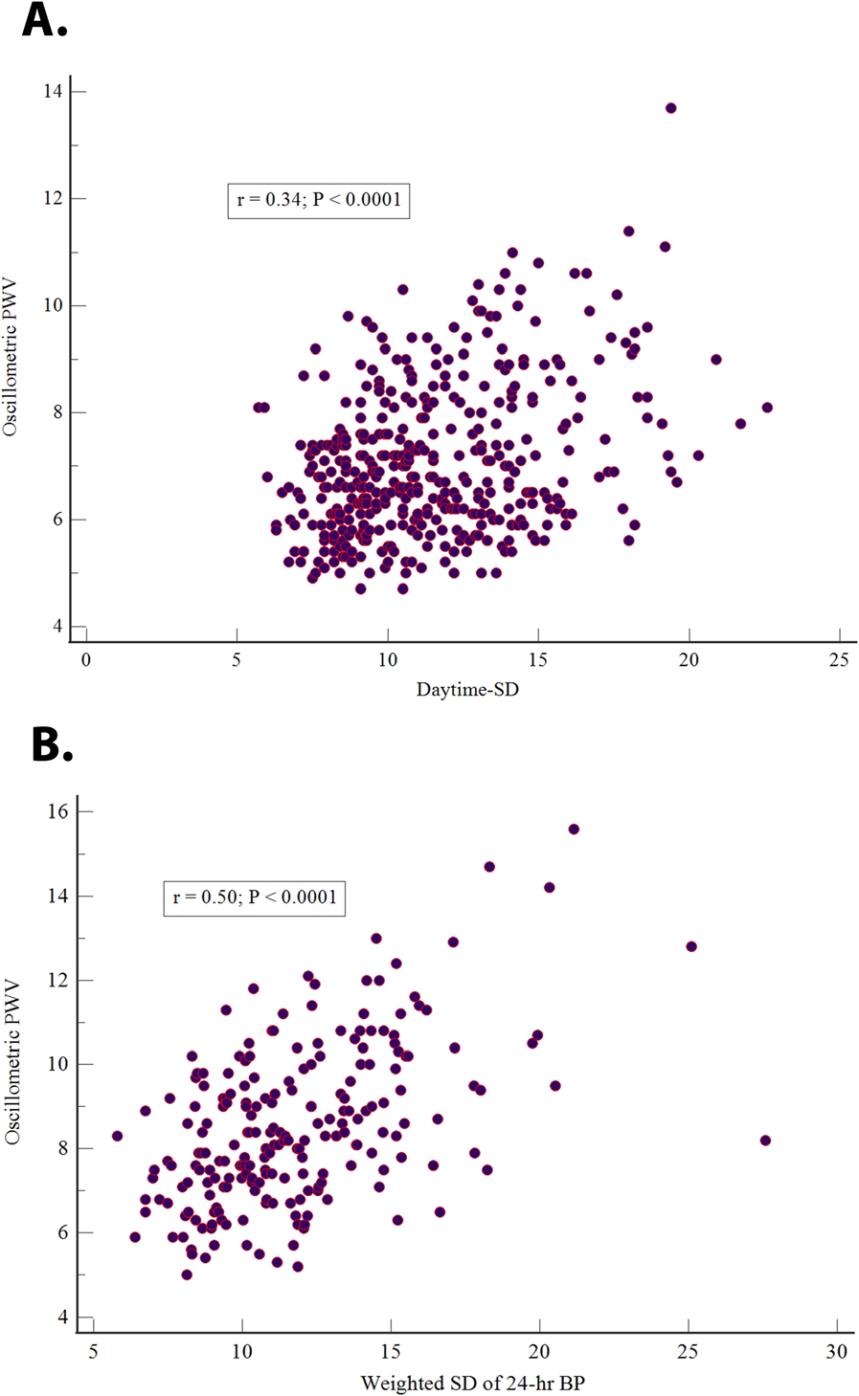
Scatter diagram of a correlation between systolic daytime-SD in suspected hypertensives (A) and weighted SD of systolic 24 hours BP in treated hypertensives (B) with Mobil-O-Graph PWV BP, blood pressure; PWV, pulse wave velocity; r, Pearson correlation coefficient; SD, standard deviation

The treated hypertension group showed similar results except for a weighted SD of 24-h, which achieved the strongest correlation followed by daytime-SD. After adjustment for age, sex, and systolic 24-h BP, all variables kept the correlation in the whole sample and suspected hypertensive group. However, daytime-SD, weighted SD of 24-h, ARV of 24-h, and SD central BP remained with a significant relation in treated patients. There was no significant difference for all comparisons of r adjusted results among the whole sample and the groups (z statistic *P* > 0.089). There was no significant difference in adjusted r in the group comparisons (z statistic *P* > 0.80). Figure 1 shows the correlation of systolic daytime-SD in the suspected group (A) and systolic weighted SD of BP 24-h in the treated group (B).

**Fig. 2:**
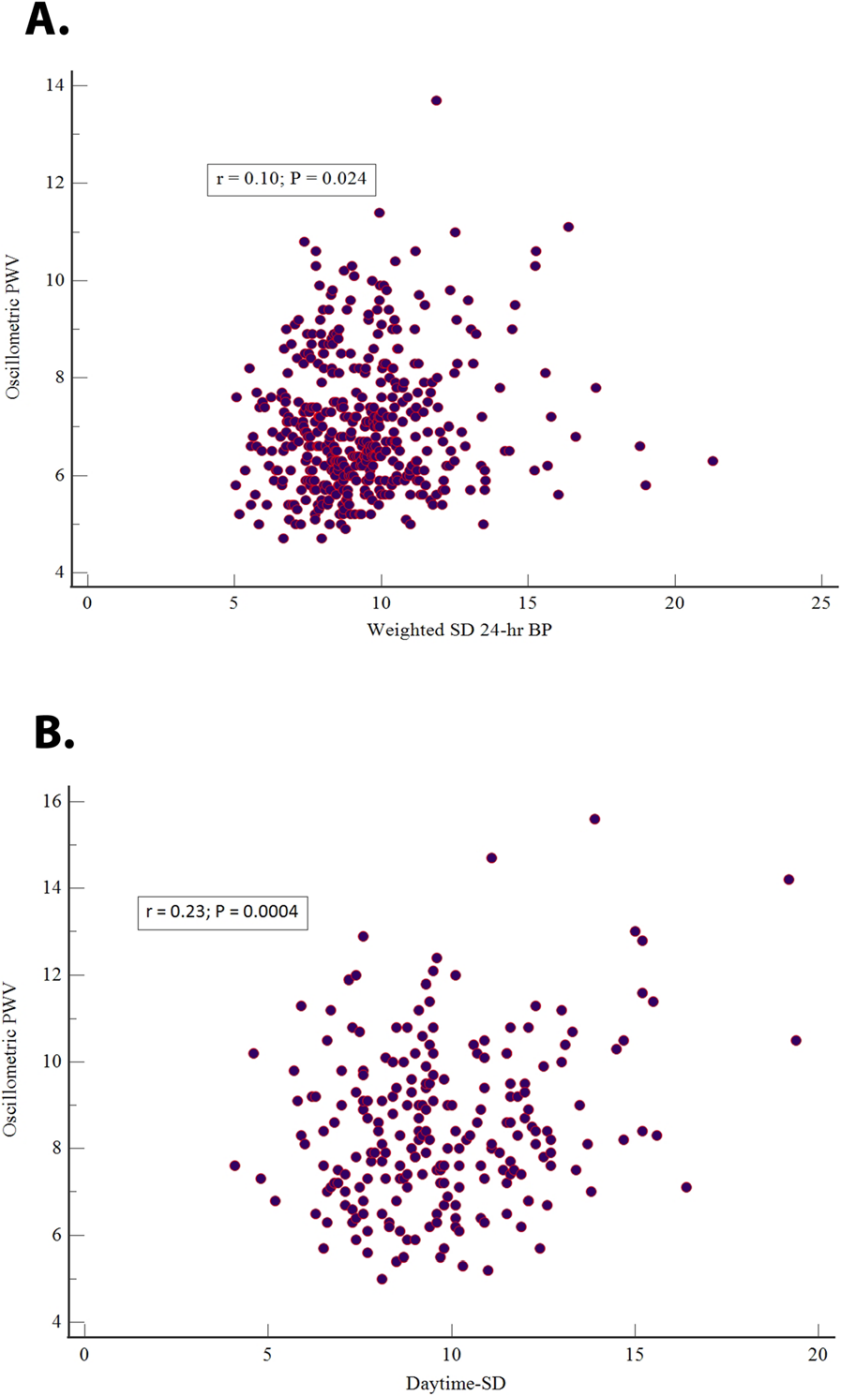
Scatter diagram of a correlation between of weighted SD of diastolic 24 hours BP in suspected hypertensives (A) and diastolic daytime-SD in treated hypertensives (B) with Mobil-O-Graph PWV BP, blood pressure; PWV, pulse wave velocity; r, Pearson correlation coefficient; SD, standard deviation

Table 3 shows data of correlation arterial stiffness with diastolic BP and BPV. In general, the univariate correlation was weaker than systolic measures. In univariate analyses, the diastolic 24-h BP in the entire sample and the suspected group and office diastolic BP in total selected did not show correlation with oPWV. After adjusted by age and sex except for diastolic 24-h BP in the treated group did not show such association. Utilizing the unadjusted analysis daytime-SD, nighttime-SD, weighted 24-h BP, ARV of 24-h, and SD of central BP showed significant relation with oPWV in the total sample. However, the nighttime-SD, weighted of 24-h BP, and SD of central BP presented such significant correlation in the suspected group. Otherwise, in treated hypertensives only SD of 24-h BP, nighttime-SD and central BP SD did not show association with Mobil-O-Graph PWV. After adjusted, only diastolic SD 24-h BP in total sample, ARV of 24-h among suspected group and SD 24-h BP, nighttime-SD, CV of 24-h BP in treated group remained without a meaningful result. The comparison after adjustment showed a difference in total sample between daytime-SD, weigthed 24-h BP, ARV of 24-h with SD of 24-h BP and CV of 24-h BP. In the suspected group by ARV 24-h with daytimeSD and weighted of 24-h BP (z statistic *P* = 0.01). In the treated hypetensives there were any difference in the results of adusted r values (z statistic *P* > 0.084). Only the correlation of ARV 24-h founded a significant difference when comparing treated with a suspected group (z statistic *P* = 0.009). Figure 2 shows the correlation of diastolic weighted SD of BP 24-h BP in a suspected group (A) and diastolic daytime-SD in the treated group (B).

**Table 3:** Correlation between oscillometric pulse wave velocity and measures of diastolic BP variability

	Pearson correlation coefficient (r)					
	Whole (649)		SH (428)		TH (221)	
	Univariable	Partial	Univariable	Partial	Univariable	Partial
**BP**		Adjusted for age and sex		Adjusted for age and sex		Adjusted for age and sex
Office BP	− 0.02^a)^	0.10^*^	0.16^**^	0.30^**^	−0.16^*^	0.20^*^
24 h-BP	− 0.05^a)^	0.10^*^	0.07^a)^	0.17^**^	−0.14^*^	0.08^a)^
**BP variability**		Adjusted for age, sex, and diastolic 24-h BP		Adjusted for age, sex, and diastolic 24-h BP		Adjusted for age, sex, and diastolic 24-h BP
SD of 24-h BP	−0.004^a)^	0.08^*^	−0.007^a)^	0.16^**^	0.10^a)^	0.10^a)^
SD of daytime BP	0.14^**^	0.21^**^	0.09^a)^	0.19^**^	0.23^**^	0.18^**^
SD of nighttime BP	0.13^**^	0.13^**^	0.13^**^	0.16^**^	0.07^a)^	0.08^a)^
Weighted SD of 24-h BP	0.14^**^	0.20^**^	0.10^*^	0.19^**^	0.22^**^	0.18^**^
ARV of 24 h	0.11^*^	0.20^**^	0.04^a)^	0.03^a)^	0.15^*^	0.24^**^
CV of 24-h BP	0.02^a)^	0.08^*^	−0.042^a)^	0.16^**^	0.19^**^	0.11^a)^
SD of central BP	0.12^*^	0.13^**^	0.13^**^	0.12^*^	0.04^a)^	0.16^*^

BP, blood pressure; r, Pearson correlation coefficient; SH, suspected hypertension; TH, treated hypertension; SD, standard deviation; ARV, average real variability; CV, coefficient of variation.

*P < 0.05, **P < 0.001, ^a)^ P, not significant

Multiple linear regression statistics reinforced the independent effects of indices of BPV on Mobil-O-Graph PWV. The systolic daytime-SD and Diastolic CV of 24-h BP were independently associated with oPWV. As well and age, systolic OBP, diastolic 24-h BP, systolic daytime, and nighttime BP (Table 4).

**Table 4:** Independent predictors of Mobil-O-Graph pulse wave velocity in a stepwise multiple regression analysis

Variable	Coefficient b_**i**_	SE	***P***-value	r _**partial**_
**Suspected hypertension**				
Age	0.09	0.001	< 0.0001	0.93
Systolic office BP	0.01	0.001	< 0.0001	0.43
Diastolic 24-h BP	− 0.02	0.003	< 0.0001	− 0.35
Systolic daytime BP	0.01	0.003	0.0003	0.17
Systolic nighttime BP	0.01	0.002	0.0001	0.19
Systolic SD of daytime	0.01	0.007	0.02	0.11
Dyslipidemia	−0.07	0.03	0.03	−0.10
**Treated hypertension**				
Age	0.11	0.003	< 0.0001	0.90
Systolic office BP	0.01	0.002	< 0.0001	0.35
Diastolic 24-h BP	−0.02	0.005	< 0.0001	−0.28
Systolic nighttime BP	0.02	0.004	< 0.0001	0.34
Diastolic CV of 24-h BP	0.03	0.01	0.02	0.15

SE, standard error; BP, blood pressure; SD, standard deviation; CV, coefficient of variation.

## Discussion

Among patients referred to record an AMBP, our study selected a population of suspected and treated hypertensives. We used the Mobil-O-Graph device to measure arterial stiffness, an operator-independent and practicable method. Our study’s main finding is to demonstrate a positively, directly, and moderate association between oPWV and systolic measures of short-time BPV. Also, oPWV showed a correlation with systolic and diastolic OBP and systolic 24-h BP. And with one measurement of central BPV. Multivariate analysis reinforced the link between oPWV and BPV and demonstrated the independence of correlation from OBP and 24-h BP.

BPV definition did not address the strength of association between BPV and arterial stiffness. The daytime-SD and wSD showed the best correlation with oPVW. But these results, when compared to the other BPV index, didn’t were significant. Daytime-SD calculates the distribution readings around a mean [21]. On the other hand, wSD measures BPV using the daytime and nighttime-SD values taking into account and separately quantifying the duration in hours of day and night. This index is independent of day-night BP fluctuations [23].

The measurement of ARV bases it on an average of variations between sequential readings. It short and dynamic changes in BPV. Hence is considered a superior index to measure short-term variability than SDs. This index shows a better correlation with cf-PWV than SDs [10]. Despite that, there is no significant difference between ARV and the other BPV measures in our results. However, we need to emphasize the sample size limited the comparisons of results between different BPV measures.

We infer the better results of SDs in univariate analyses, although no significant, could be based on the method chosen to measure arterial stiffness. Mobil-O-Graph PWV is highly dependent on age and BP values. So, we could presuppose this difference reflects the best association between SDs variability measures and systolic BP. Still, it seems this is not the case because the results showed independence on 24-h BP. Then we can’t explain these differences. And this question claims for other studies.

BPV represents a heterogeneous phenomenon that strongly depends on the estimation method and the period evaluated [24]. Indeed, four previous studies have shown that greater BPV is associated with greater arterial stiffness. Two studies presented limitations due to lack of adjustment for antihypertensive medication or excluded individuals on elevated BP medication [25, 26]. On the Multiethnic Study of Atherosclerosis (MESA) among individuals free of CVD and not taking antihypertensives, the authors showed an association between long-term systolic BPV and a 10-year change in arterial stiffness independent of mean BP level [27]. From data collected in 2 large separate populations, Schilacci et al. [10] demonstrated an independent, moderate relation between short-term variability of 24-h systolic BP and aortic stiffness in hypertension. All of the measures of short-term systolic BPV were consistently and directly related to cf-PWV. ARV of 24-h systolic BP had the most substantial relation with cf-PWV, followed by wSD.

Unlike other studies using cf-PWW [10], the diastolic BPV measures showed a positive correlation with oPWV. However, it was weaker than founded with systolic BPV. Here, after analyzing the changes in results after adjustment. The correlation between oPWV and diastolic BP measures seems dependent on BP values. Similarly, the apparent best association in treated than suspected hypertensive. Also, look dependent on BP differences between groups in treated than suspected hypertensive. Also, look dependent on BP differences between groups.

It is worth commenting on the mechanisms behind this association. Due to the design of our study, we cannot accurately explain this relationship. Aging, BP, and arterial stiffness are closely related. And this association might help explain the results. Nevertheless, the raising in arterial stiffness with aging might be a cause or consequence of raising in BP hence in BPV. Aging increases arterial stiffness, which increases BP, and pressure variability is well established [28, 29].

Baroreceptor function is a significant determinant of BPV. Among young and middle-aged hypertensive individuals. Arterial remodeling and reduce central arterial vessel compliance impairs baroceptor sensitivity. However, a reduction in baroreceptor sensibility accompanies an increase in BP and aging. It was not an independent determinant of the relationship between BPV and baroreceptor function. Thus, more studies are necessary to investigate the causality of this association. Therefore, more tasks are required to examine the basis of this association [30].

Our study presents some force—all PWV measurements performed on the same day, arm, and equivalent cuff size as ABPM records. Mobil-O-Graph device permits a measure operator-independent. Also, we considerer only BPV measures calculate from ABPM. All 24-h BP ambulatory recordings included for analysis fulfilled high-quality criteria. It was important to sort the sample into suspected and treated hypertensive, as demonstrated by a remarkable study [10].

Indeed, the study shows some limitations. Using Mobil-O-Graph to measure PWV also has a negative aspect. Age and BP importantly determine Mobil-O-Graph PWV. We cannot say with certainty if what we measure is arterial stiffness—the size sample limited comparisons of association with oPWV with different BPV measures. And, there were significant differences among demographic and clinical features between the groups studied. Even if the results showed an association between central BPV, we believe that the limited numbers of central measurements underestimated SD central BP, impacting the association’s strength. Our study is cross-sectional, thus precludes conclusions about causality relations between Mobil-O-Graph PWV and BPV.

Estimated cf-PWV calculated from age and media BP predicted major CV events independently of Systematic COronary Risk Evaluation (SCORE), risk factors, and cf-PWV [31]. Mobil-O-Graph permits easily measure PWV and the variability of BP at the same time, using one equipment. It opens the prospect for longitudinal studies to establish how much these measures impact each other, their relationship with target organ injuries, and even CV outcomes. Then, longitudinal studies to set if these measures would use as therapeutic targets in the future.

## Conclusion

Our study has demonstrated a positive and independent association between Mobil-O-Graph PWV and BPV measures. This correlation was moderate to systolic BPV and only weak to diastolic BP.

## Data Availability

The datasets used and/or analysed during the current study are available from the corresponding author on reasonable request.

## Abbreviations

ABPM: Ambulatory blood pressure monitoring
AMBP: Ambulatory monitoring blood pressure
ARV: Average real variability
BP: Blood pressure
BPV: Blood pressure variability
CKD: Chronic kidney disease
CV: Cardiovascular risks
CVD: Cardiovascular disease
CV 24-h: Coefficient of variation of 24-h BP
cf-PWV: Carotid-femoral pulse wave
Daytime-SD: Standard deviation of daytime BP
DBP: Diastolic blood pressure
Nighttime-SD: Standard deviation of nighttime blood pressure
oPWV: Oscillometric pulse wave velocity
PWV: Pulse wave velocity
r: Pearson correlation coefficient
SH: suspected hypertension
SBP: Systolic blood pressure
SD: Standard deviation
SE: Standard error
24 h-SD: Standard deviation of 24 h blood pressure
TH: treated hypertension
wSD: Weighted SD of 24-h BP
